# Irreversible Inhibition of Glutathione S-Transferase by Phenethyl Isothiocyanate (PEITC), a Dietary Cancer Chemopreventive Phytochemical

**DOI:** 10.1371/journal.pone.0163821

**Published:** 2016-09-29

**Authors:** Vandana Kumari, Marzena A. Dyba, Ryan J. Holland, Yu-He Liang, Shivendra V. Singh, Xinhua Ji

**Affiliations:** 1 Center for Cancer Research, National Cancer Institute, Frederick, Maryland, United States of America; 2 Basic Science Program, Leidos Biomedical Research, Inc., Frederick National Laboratory for Cancer Research, Frederick, Maryland, United States of America; 3 Department of Pharmacology and Chemical Biology and University of Pittsburgh Cancer Institute, University of Pittsburgh School of Medicine, Pittsburgh, Pennsylvania, United States of America; University of Texas Health Science Center at Houston, UNITED STATES

## Abstract

Dietary isothiocyanates abundant as glucosinolate precursors in many edible cruciferous vegetables are effective for prevention of cancer in chemically-induced and transgenic rodent models. Some of these agents, including phenethyl isothiocyanate (PEITC), have already advanced to clinical investigations. The primary route of isothiocyanate metabolism is its conjugation with glutathione (GSH), a reaction catalyzed by glutathione S-transferase (GST). The pi class GST of subunit type 1 (hGSTP1) is much more effective than the alpha class GST of subunit type 1 (hGSTA1) in catalyzing the conjugation. Here, we report the crystal structures of hGSTP1 and hGSTA1 each in complex with the GSH adduct of PEITC. We find that PEITC also covalently modifies the cysteine side chains of GST, which irreversibly inhibits enzymatic activity.

## Introduction

Epidemiological support for a protective role of cruciferous vegetables against cancer risk reduction is quite persuasive [1]. For example, a very recent meta-analysis of four case-control reports and an equal number of cohort studies suggested a significant inverse association between cruciferous vegetable consumption and the risk of ovarian cancer [2]. Cancer preventive phytochemicals in widely consumed cruciferous vegetables (*e*.*g*., watercress, cabbage, broccoli, mustard, and so forth) are stored as thioglucoside conjugates (glucosinolates) that are enzymatically hydrolyzed to bioactive isothiocyanates upon cutting or chewing of the plant through catalytic mediation of myrosinase [3]. Phenethyl isothiocyanate (PEITC) is one of the best studied members of the isothiocyanate family with a fairly strong pre-clinical evidence for its cancer preventive efficacy [4]. Besides cancer prevention, intragastric administration of PEITC suppressed acetaminophen metabolism and consequently hepatotoxicity in mice [5]. The bench-bedside translation of some of these exciting pre-clinical observations is in progress as evidenced by completed or ongoing trials listed in the clinicaltrials.gov (*e*.*g*., NCT00691132, NCT01790204).

**Fig 1:**
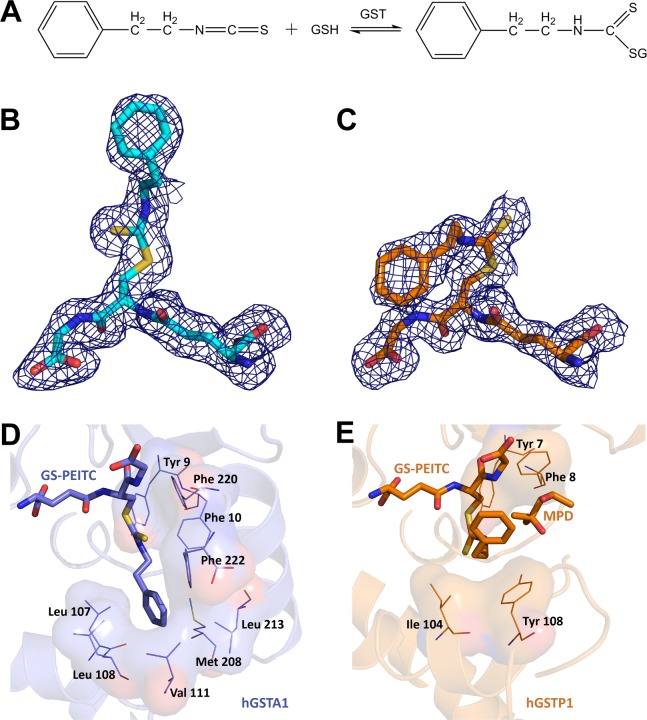
Distinct Binding Modes of GS-PEITC in GST Enzymes. (**A**) GST catalyzes the conjugation of PEITC with GSH in both forward (formation of GS-PEITC) and reverse (dissociation of GS-PEITC) directions. (**B,C**) The GS-PEITC adduct bound in the active site of hGSTA1 and hGSTP1, respectively, is illustrated as a stick model in atomic color scheme (nitrogen in blue, carbon in cyan (hGSTA1) or orange (hGSTP1), oxygen in red, and sulfur in yellow) outlined with annealed omit map (blue mesh, *F*_o_-*F*_c_ electron density contoured at 2.0 σ). Note the distinct conformations of GS-PEITC in the two GST active sites. (**D**) In the hGSTA1:GS-PEITC structure (PDB entry 5JCU), the phenethyl moiety of GS-PEITC docks into a hydrophobic pocket formed by the side chains of Leu107, Leu108, Val111, Met208, Leu213, Phe222, Phe10 and Phe220. (**E**) In the hGSTP1:GS-PEITC structure (PDB entry 5JCW), however, it points toward the solvent channel, stacking with the hydrophobic side chains of Ile104 and Tyr108 and in contact with a 2-methyl-2,4-pentanediol (MPD) molecule from cryoprotectant. In panels D and E, ligand molecules are illustrated as thick sticks, amino acid residues as thin sticks outlined with their surfaces, and protein structures as ribbon diagrams in the background. Nitrogen atoms are shown in blue, oxygen in red, and sulfur in yellow. Carbon atoms are shown in blue in hGSTA1:GS-PEITC, but in orange in hGSTP1:GS-PEITC.

Glutathione *S*-transferases (GST) are involved in the metabolism of PEITC by catalyzing its conjugation with glutathione (GSH, Fig 1A) [6]. Because of GSH conjugation, it is not surprising that the plasma half-life (*T*_max_) of PEITC is very short [7,8]. In the metabolism of PEITC, the pi class GST isoform of subunit type 1 (hGSTP1) is relatively more efficient than the alpha class GST isoform of subunit type 1 (hGSTA1) [9]. Next-generation PEITC analogues with improved pharmacokinetic behavior (*i*.*e*., increased plasma and tissue levels and/or reduced clearance) and therapeutic efficacy is therefore warranted to increase its efficacy for cancer prevention. Chemical refinement of PEITC for improvement, however, is contingent upon determination of the crystal structure of the GST isoforms in complex with the GSH conjugate of PEITC (GS-PEITC) to identify amino acids critical for catalysis. Here, we report the crystal structures of hGSTP1 and hGSTA1 in complex with GS-PEITC (hGSTP1:GS-PEITC and hGSTA1:GS-PEITC, respectively). These structures not only define the protein-product interactions at atomic resolution, but also reveal that PEITC covalently modifies cysteine side chains in GST and thereby irreversibly inhibits enzymatic activity.

## Materials and Methods

### Protein expression and purification

The hGSTP1 was prepared as described [10,11] and concentrated to 21.1 mg/mL for crystallization. The hGSTA1 was prepared as described [11,12] and concentrated to 13 mg/mL for crystallization.

### Crystallization and X-ray diffraction

GSH and PEITC were purchased (Sigma-Aldrich) and stock solutions were made in water containing 10 mM dithiothreitol (DTT). Crystals of the hGSTP1:GS-PEITC complex were grown by hanging drop vapor diffusion. The hGSTP1 protein (21.1 mg/ml), GSH (63 mM) and PEITC (120 mM) were mixed at a ratio of 10:1:1 and the mixture was incubated for 6 h at 4°C before setting up for crystallization. Initial crystallization conditions were obtained from PEG screening kit (Qiagen) using Hydra II Plus One (Matrix Technologies Corporation) crystallization robot and optimized further. The 1 μL drops were equilibrated at room temperature (RT) against 4 μL well solution containing 20% (w/v) PEG-8000 and 0.2 M Ca(OAc)_2_ in 0.1 M MES buffer (pH 6.0). Plate-shaped crystals appeared in a few h and reached to final size (0.2 x 0.2 x 0.05 mm^3^) in 1–2 days. The crystals were cryoprotected in the well solution mixed with 20% 2-methyl-2,4-pentanediol (MPD), 6.3 mM GSH, and 12.0 mM PEITC.

Crystals of the hGSTA1:GS-PEITC complex were grown by sitting drop vapor diffusion. The hGSTA1 protein (13 mg/ml), GSH (63 mM), and PEITC (120 mM) were mixed at a ratio of 10:1:1 and the mixture was incubated for 6 h at 4°C before setting up for crystallization. The crystals were obtained from PEG screening kit (Qiagen) using Hydra II Plus One (Matrix Technologies Corporation) from G3 condition (20% (w/v) PEG 3350, 0.2 M sodium acetate) by mixing 0.3 μL protein and 0.1 μL well solution. Rod shaped crystals appeared in one day and reached to final size (0.3 x 0.1 x 0.1 mm^3^) in 2–3 days. The crystals were cryoprotected in the well solution mixed with 20% ethylene glycol, 6.3 mM GSH, and 12.0 mM PEITC.

X-ray diffraction data were collected from a single crystal for each complex at the SER-CAT 22-BM beam line of the Advanced Photon Source at Argonne National Laboratory and processed using the HKL3000 program suite [13]. Data collection statistics are summarized in Table 1.

**Table 1:** Crystallographic Data Collection and Refinement Statistics.

	hGSTP1:GS-PEITC	hGSTA1:GS-PEITC
**PDB ID code**	5JCW	5JCU
**Data collection**		
X-ray source	APS SER-CAT 22-BM	APS SER-CAT 22-BM
Space group	*C*2	*C*2
Unit cell parameters		
*a* (Å)	78.37	100.34
*b* (Å)	88.94	92.67
*c* (Å)	69.48	104.05
*β* (°)	97.85	92.18
Wavelength (Å)	1.0	1.0
Resolution (Å)	50–1.95 (2.02–1.95) ^a^	30–1.93 (2.00–1.93)
*R*_merge_^b^ (%)	0.089 (0.54)	0.083 (0.866)
<*I*/σ(*I*)>	17.11 (2.21)	18.04 (1.63)
Completeness (%)	99.8 (98.7)	99.2 (98.4)
Average multiplicity	4.7 (4.3)	4.9 (4.9)
**Refinement**		
Resolution (Å)	35.984–1.945 (2.047–1.945)	29.802–1.925 (2.026–1.925)
No. of reflections for *R*_work_	33544 (4657)	71262 (9780)
No. of reflections for *R*_free_	1000 (139)	998 (123)
*R*_work_	15.60	17.72
*R*_free_	19.41	24.00
Number of non-H atoms		
Protein	3300	7221
Water	479	1100
Other entities	125	216
*B* values (Å^2^)		
Overall	26.326	33.165
Protein	24.981	32.236
tdWater	34.088	37.720
Other entities	32.10	41.038
R.m.s.^c^ deviations		
Bond lengths (Å)	0.006	0.009
Bond angles (°)	0.965	1.090
Ramachandran plot		
Favored (%)	97.14	96.27

^a^Values in parentheses are for the highest resolution shell.

^b^*R*_merge_ = Σ_*hkl*_ Σ_*i*_|*І*_*i*_ (*hkl*) ‒ <*І* (*hkl*)>|/Σ_*hkl*_ Σ_*i*_ *І*_*i*_ (*hkl*).

^c^Root-mean-square.

### Crystal structure determination

The structures were determined by Fourier synthesis. The starting model for hGSTP1 was the 1.9-Å structure of hGSTP1 (Protein Data Bank (PDB) ID: 3PGT) [14] with ligand and solvent molecules removed. The starting model for hGSTA1 was the 2.6-Å structure of hGSTA1 (PDB ID: 1GUH) [15] with ligand and solvent molecules removed. The initial *F*_o_-*F*_c_ map revealed positive electron density for GS-PEITC and other ligands. Models were manually adjusted using the program COOT [16]. Conventional *R*-factors (*R*_work_ and *R*_free_) were used to monitor the progress of refinement using the Phenix package [17]. Water molecules were located and incorporated at later stages of the refinement. Careful inspection of the 2*F*_o_-*F*_c_ and *F*_o_-*F*_c_ maps revealed the Cys112-PEITC adduct formation in hGSTA1. Refinement statistics are summarized in Table 1. Coordinates and structure factors have been deposited with the PDB under the accession codes 5JCU and 5JCW.

### High-resolution mass spectrometry

High-resolution mass spectrometry (MS) experiments were performed at the Biophysics Core Facility in the Structural Biophysics Laboratory, Center for Cancer Research, National Cancer Institute. To identify protein modifications, the molecular weight of hGSTP1 and hGSTA1 proteins, either unmodified or unmodified, was obtained first. Then, the peptide mass mapping method was used to characterize the protein further. For molecular weight analysis, 1 mg/mL protein was mixed with 300 *μ*M GSH and 80 *μ*M PEITC and incubated for 30 min at room temperature (RT). For peptide mapping, hGSTP1 and hGSTA1 were cleaved into smaller peptides using sequencing grade chymotrypsin (Promega) and trypsin (Promega), respectively. 6 M urea in 100 mM Tris buffer was added to the protein sample. The sample was reduced for 1 h at RT by mixing 10 mM DTT followed by alkylating the sample by 40 mM iodoacetamide for 1 h at RT. Unreacted iodoacetamide was consumed by adding 20 mM DTT for 1 h at RT. Urea concentration was reduced to 0.6 M by adding water. Protein sample was digested overnight at 37°C by adding 100 *μ*L sequencing grade solution containing 20 *μ*g chymotrypsin. The reaction was stopped by adjusting the pH to lower than 6 using concentrated acetic acid. The digest was analyzed using high-resolution MS coupled with liquid chromatography (LC).

MS data were acquired on an Agilent 6520 Accurate-Mass Q-TOF LC/MS System (Agilent Technologies, Inc., Santa Clara, CA) equipped with a dual electro-spray source and operated in the positive-ion mode. Separation of intact proteins was performed on a Poroshell 300SB-C3 column (2.1 mm x 75 mm, particle size 5.0 *μ*m) and of digested peptides on a Poroshell 120SB-C18 column (2.1 mm x 75 mm, particle size 2.7 *μ*m). In peptide mapping experiments, the analytes were eluted at a flow rate of 0.2 mL/min with a 5 to 90% organic gradient over 22 min and holding organic for 5 min. Both mobile phases, water and acetonitrile, contained 0.1% formic acid. The instrument was used in a full-scan time-of-flight (TOF) mode or product ion scan (MS/MS) mode. MS source parameters were set with a capillary voltage of 4 kV, the fragmentor voltage of 190 V and skimmer of 65 V. The gas temperature was 350°C, drying gas flow was 10 L/min and nebulizer pressure was 30 psig. Nitrogen was used as a collision gas. Data were acquired at high resolution (3,200 m/z) mode at 4 GHz. TOF-MS mass spectra were recorded across the range 100–3,200 m/z. Q-TOF-MS/MS experiments were carried out in the range 100–2000 m/z with a scan rate of 4 spectra/s and they were accomplished with collision energy of 72 V. To maintain mass accuracy during the run time, an internal mass calibration sample was infused continuously during the LC/MS runs. Data acquisition was performed using Mass Hunter Workstation (version B.02.00). For data analysis of mass spectra, the Mass Hunter Qualitative Analysis software (version B.03.01) with Bioconfirm Workflow was used. The software provides maximum entropy deconvolution algorithm to calculate intact protein molecular weights and molecular feature extraction algorithm, which finds peptides and determines their masses via resolved isotope deconvolution.

The list of peptide masses was correlated to the protein amino acid sequence with modification applied to specific amino acids. The matching rules included predicted modification such as carbamylation, deamination, pyroGlu, and oxidation. In targeted MS/MS analysis, the peptide, which carried modification, was fragmented by collision-induced dissociation.

### GST catalytic activity assay

Kinetic experiments were performed at 37°C using a standard UV-visible spectrophotometer. GSH (200 *μ*M) was added to a solution containing either 5 *μ*g of hGSTP1 or hGSTA1 in 0.1 M phosphate buffer (pH 6.6). After the GSH-containing buffer and enzyme reached thermal equilibrium, each solution was treated with either an ethanolic solution of PEITC (400 *μ*M) or ethanol as a control and incubated for 30 min. Enzyme activity was probed with CDNB (200 *μ*M) which was added directly to the reaction mixture. In each experiment the data were analyzed at 340 nm and the rate was derived by fitting the data to an exponential curve typical for first order processes.

## Results and Discussion

### Distinct Binding Modes of GS-PEITC in GST Enzymes

We determined the crystal structures of the hGSTA1:GS-PEITC and hGSTP1:GS-PEITC complexes at 1.93- and 1.95-Å resolution, respectively (Table 1). The structures show that the orientation of the phenethyl moiety of the conjugate was significantly different in the two GST enzymes (Fig 1B and 1C). In the hGSTA1:GS-PEITC structure, the phenethyl moiety of GS-PEITC docks into a hydrophobic pocket formed by the side chains of Leu107, Leu108, Val111, Met208, Leu213, Phe222, Phe10 and Phe220 (Fig 1D). In the hGSTP1:GS-PEITC structure, however, it points toward the solvent channel, stacking with the hydrophobic side chains of Ile104 and Tyr108 (Fig 1E). The binding pocket for the phenethyl moiety of GS-PEITC in hGSTP1 can accommodate more diverse and bigger ligands than that in hGSTA1. The detailed structural information would guide the modification of the structure of PEITC aiming to avoid its conjugation with GSH.

The enzymatic activity of GST is not a direct mechanism how PEITC prevents cancer. Many environmental carcinogens require activation through cytochrome P450 enzymes. As reviewed by us previously, PEITC has the ability to inhibit cancer initiation if administered prior to the carcinogen exposure as well as post-initiation cancer progression due to apoptosis induction [18]. It has also been shown that the protective action of PEITC against acetaminophen (APAP) toxicity is attributed to the blocking of APAP activation through inhibition of P450 enzymes [5].

PEITC is a Michael acceptor, and its chemical reactivity with cellular nucleophiles like GSH is expected. As previously reported, it causes depletion of cellular GSH [19]. The present study is the first to clearly demonstrate covalent modification of cysteine residues in both hGSATA1 and hGSTP1, which also explains the mechanism for inhibition of GST activity by PEITC treatment.

### Covalent modification of Cys112 in hGSTA1 by PEITC

**Fig 2:**
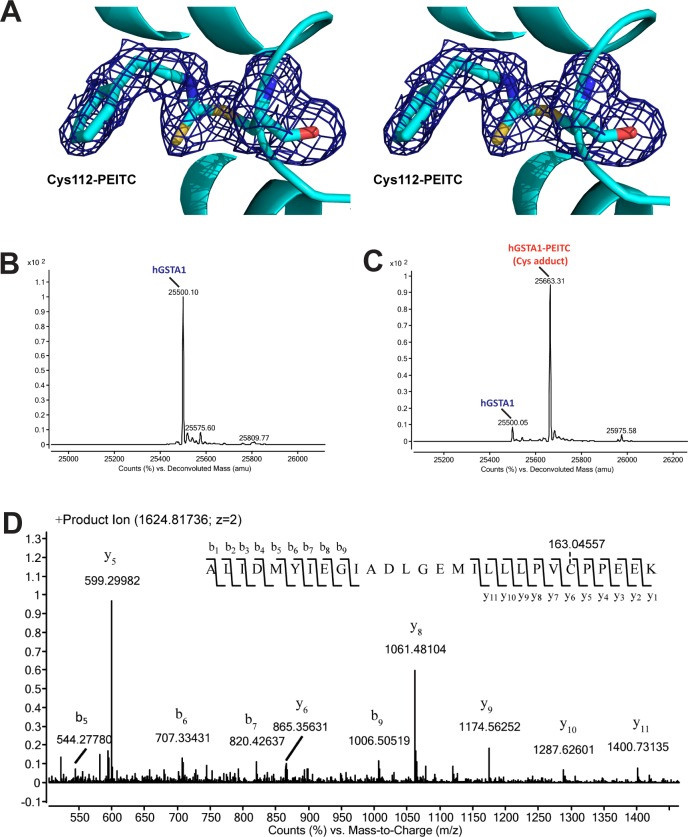
Cys112 adduct of PEITC in hGSTA1. (**A**) In hGSTA1, the side chain of Cys112 was covalently modified by PEITC. The Cys112-PEITC adduct is illustrated in stereo as a stick model in atomic color scheme (nitrogen in blue, carbon in cyan, oxygen in red, and sulfur in yellow) along with annealed omit map (blue mesh, *F*_o_-*F*_c_ electron density contoured at 2.0 σ) of the modified residue. The ribbon diagram of hGSTA1 (helices as spirals and loops as tubes) is shown. (**B**) Deconvoluted mass spectrum for unmodified hGSTA1. (**C**) Deconvoluted mass spectrum for modified hGSTA1 by PEITC (hGSTA1-PEITC). (**D**) Product ion spectrum of doubly charged precursor of m/z 1624.81736. The modification site at Cys112 of hGSTA1 within the tryptic peptide ALIDMYIEGIADLGEMILLLPVC*PPEEK was identified on the basis of singly charged b- and y-ion series observed on the product-ion spectrum and the mass difference, corresponding to the mass of PEITC.

**Fig 3:**
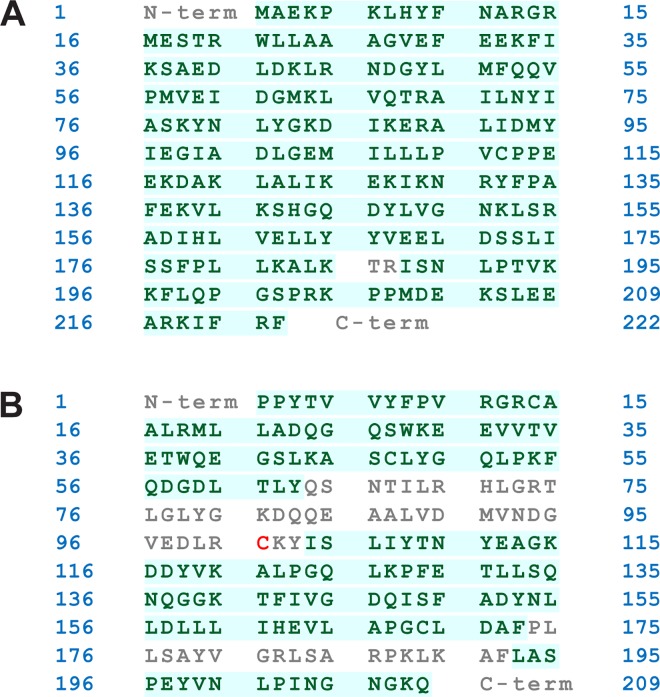
Amino acid sequence coverage of modified proteins. (**A**) hGSTA1 and (**B**) hGSTP1. In peptide mass mapping, covered amino acid residues are shown in green; noncovered in grey, including a cysteine (in red).

The hGSTA1 contains a single cysteine residue, Cys112. The hGSTA1:GS-PEITC structure reveals that the side chain of Cys112 was covalently modified by PEITC (Fig 2A). The PEITC modification was also elucidated using high-resolution mass spectrometry (MS). First, the molecular weight obtained for unmodified (Fig 2B) and modified (Fig 2C) hGSTA1 was found to differ by the mass of PEITC (163 Da). Second, the proteolytic peptide masses were matched with the hGSTA1 sequence with PEITC modification applied to Cys112 (peptide mapping, Fig 3A and Table 2). Third, the exact site of modification was identified by the tandem MS (MS/MS) analysis as Cys112 (Fig 2D). Like the sulfhydryl group of GSH, the sulfhydryl group of Cys112 acts as a nucleophile and attacks the isothiocynate central C atom of PEITC and forms an irreversible adduct with hGSTA1 (hGSTA1^Cys112-PEITC^).

**Table 2:** List of modified peptides with sequences identified by the BioConfirm software.

Protein	Seq Loc	Mass	Sequence	Mods	Pred Mods
**hGSTA1**	A(90–117)	3247.62015	ALIDMYIEGIADLGEMILLLPVCPPEEK	PEITC(A112)	
**hGSTP1**	A(39–55)	2160.98052	QEGSLKASCLYGQLPKF	PEITC(A47)	3*Carbamylation(+43.005814); 1*Deamidation(+0.984016)A54A44 A^A39
	A(154–173)	2342.18212	NLLDLLLIHEVLAPGCLDAF	PEITC(A169)	1*Deamidation(+0.984016)A154
	A(8–38)	3841.869	FPVRGRCAALRMLLADQGQSWKEEVVTVETW	PEITC(A14)	2*Deamidation(+0.984016); 2*Carbamylation(+43.005814); 1*Oxidation(+15.994915)A24A26 A29A^A19

### Covalent modification of Cysteines in hGSTP1 by PEITC

**Fig 4:**
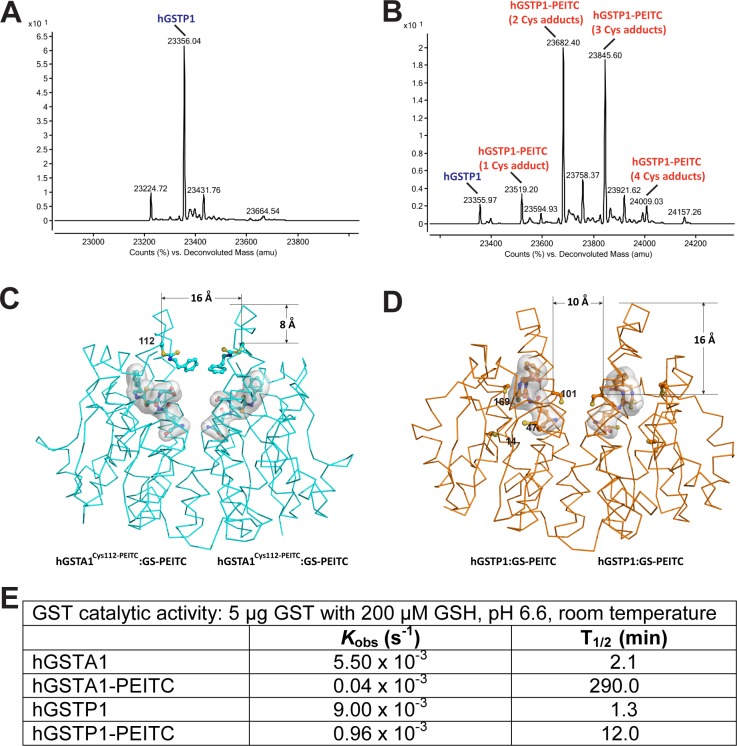
Impact of cysteine modification on the catalytic activity of GST. (**A**) Deconvoluted mass spectrum for unmodified hGSTP1. (**B**) Deconvoluted mass spectrum for hGSTP1-PEITC. (**C**) Dimer of hGSTA1^Cys112-PEITC^:GS-PEITC indicates that the formation of C112-PEITC introduces significant disturbance at the subunit interface. The hGSTA1 is illustrated as a Cα trace and the Cys112-PEITC adduct is shown as a ball-and-stick model. The GS-PEITC product is shown as a stick model outlined with a molecular surface. Atomic color scheme: carbon in cyan, nitrogen in blue, oxygen in red, and sulfur in yellow. The distances in Å are measured from Cα positions. (**D**) Dimer of hGSTP1:GS-PEITC shows the location of four cysteine residues (Cys14, Cys47, Cys101, and Cys169), among which the Cys101 pair is located in the solvent channel. The cysteine side chains are shown as ball-and-stick models. Atomic color scheme: carbon in orange, nitrogen in blue, oxygen in red, and sulfur in yellow. (**E**) Catalytic activity for unmodified and modified GST enzymes, showing the inhibition of GST activity by PEITC modification of the enzyme. The reaction rate was derived by fitting the data to an exponential curve typical for first order processes.

Unlike hGSTA1 that contains only one cysteine residue, hGSTP1 contains four (Cys14, Cys47, Cys101, and Cys169). High-resolution MS showed that the modification of these cysteine side chains by PEITC resulted in multiple species of modified hGSTP1 proteins, including 1-, 2-, 3, and 4-Cys adducts (Fig 4A and 4B). Peptide mapping experiment confirmed the presence of modified cysteine residues 14, 47, and 169 (Fig 3B and Table 2). Therefore, unmodified and many species of modified hGSTP1 proteins were present in the crystal lattice at the same time, none of which was as abundant as could be revealed by observable electron density. As expected, the hGSTP1:GS-PEITC structure did not show any modified cysteine side chain.

As phase II detoxification enzymes, cytosolic GSTs exist and function as stable homo- or heterodimers. The dimer is required to maintain the functional conformations at the GSH-binding site and xenobiotic substrate-binding site in each subunit. Although a stable monomer of hGSTP1 could be created, it was catalytically inactive [20]. The two subunits of a murine alpha class GST were found to exhibit negative cooperativity and that the disruption of signaling between the two subunits released such cooperativity [21]. As aforementioned, Cys112 is the only cysteine residue in the sequence of hGSTA1. In the hGSTA1 dimer, the Cys112 pair is located near the entrance of the solvent channel at the subunit interface (Fig 4C). One can imagine that the covalent modification of both Cys112 side chains with PEITC must have significant impact on the dynamics of the interface, the openness of the solvent channel, the binding of GSH and substrate, and the release of the product. Indeed, the formation of Cys112-PEITC in hGSTA1 did alter the catalytic activity significantly, leading to more than two orders of magnitude inhibition in vitro. In contrast, the PEITC treatment of hGSTP1 resulted in less than one order of magnitude inhibition of catalytic activity (Fig 4E).

Among the four cysteine pairs in the hGSTP1 dimer, only the Cys101 pair is located in the solvent channel at the subunit interface. Unlike the Cys112 pair in the hGSTA1 dimer, which is located near the entrance of the solvent channel, the Cys101 pair in the hGSTP1 dimer is located much deeper and hence near the bottom of the channel (Fig 4C and 4D). Because the solvent channel becomes much narrower near the bottom, there is not enough room for the covalent modifications of both Cys101 side chains at the same time. With a single Cys-PEITC near the bottom of the solvent channel, the protein dynamics, substrate and cofactor binding, and product release may not be affected significantly. Assuming the four cysteine side chains have equal opportunities for modifications by PEITC (Fig 4B), the amount of Cys101-modified hGSTP1 may reach 1/8 of the total hGSTP1 population. In reality, however, it is not even one eighth because Cys101 is less reactive than Cys47 for covalent modification. For example, Cys47 is the target for covalent reaction with the diuretic drug ethacrynic acid (EA) [22]. In the absence of Cys47, Cys101 became a target for modification by EA[23]. Therefore, the population of hGSTP1 containing Cys101-PEITC must be much less than one eighth. This estimation of Cys101-modified hGSTP1 population is consistent with our in vitro data showing that the PEITC treatment resulted in about one order of magnitude inhibition of enzymatic activity (Fig 4E).

## Conclusions

We have obtained the first and direct evidence that PEITC forms covalent adducts with cysteine residues of GST, which irreversibly inhibits the catalytic activities of the enzyme, demonstrating that PEITC is able to suppress its own metabolism. The effect of inhibition, however, is more pronounced toward hGSTA1. Our structural and functional data also provide molecular basis for previous observations that hGSTP1 is more efficient than hGSTA1 in catalyzing the conjugation of GSH with PEITC [9]. Because PEITC is an effective inhibitor for hGSTA1-catalyzed GSH conjugation of PEITC, we do not need to worry about hGSTA1 any further in the effort of creating next-generation PEITC analogues with improved pharmacokinetic behavior, especially reduced clearance, for cancer prevention. Chemical refinement of PEITC for improvement toward hGSTP1 is now undertaken on the basis of the hGSTP1:GS-PEITC structure that reveals PEITC-hGSTP1 interactions at high resolution. We will try to make the molecules not only less suitable for GSH conjugation by hGSTP1, but also more suitable for Cys101 modification of hGSTP1.

The present study provides structural data necessary to conceptualize synthesis of next generation PEITC analogs. PEITC itself is generally safe based on aforementioned preclinical and clinical data. Whether or not reduced metabolism of PEITC analogs due to evasion of GSH conjugation and possibly increased plasma level will require detailed pharmacokinetic and toxicology studies. When the synthesis of such PEITC analogs is achieved, their toxicity or biological activity (cancer preventive effect) will be elucidated.
